# At-risk registers integrated into primary care to stop asthma crises in the UK (ARRISA-UK): study protocol for a pragmatic, cluster randomised trial with nested health economic and process evaluations

**DOI:** 10.1186/s13063-018-2816-z

**Published:** 2018-08-29

**Authors:** Jane R. Smith, Stanley Musgrave, Estelle Payerne, Michael Noble, Erika J. Sims, Allan B. Clark, Garry Barton, Hilary Pinnock, Aziz Sheikh, Andrew M. Wilson, On behalf of the ARRISA-UK Group, Polly-Anna Ashford, Chris Butler, Ann-Louise Caress, Chris Griffiths, Lisa Irvine, Martin Pond, David Price, Ann Marie Swart, Susan Stirling, Mike Thomas, Samantha Walker

**Affiliations:** 1 University of Exeter, Exeter, UK; 2 University of East Anglia, Norwich, UK; 3 Acle Medical Centre, Norwich, UK; 4 University of Edinburgh, Edinburgh, UK

**Keywords:** At-risk asthma, General practice, Pathway of care, Register, eLearning, Hospitalisation

## Abstract

**Background:**

Despite effective treatments and long-standing management guidelines, there are approximately 1400 hospital admissions for asthma weekly in the United Kingdom (UK), many of which could be avoided. In our previous research, a secondary analysis of the intervention (ARRISA) suggested an improvement in the management of at-risk asthma patients in primary care. ARRISA involved identifying individuals at risk of adverse asthma events, flagging their electronic health records, training practice staff to develop and implement practice-wide processes of care when alerted by the flag, plus motivational reminders. We now seek to determine the effectiveness and cost-effectiveness of ARRISA in reducing asthma-related crisis events.

**Methods:**

We are undertaking a pragmatic, two-arm, multicentre, cluster randomised controlled trial, plus health economic and process evaluation. We will randomise 270 primary care practices from throughout the UK covering over 10,000 registered patients with ‘at-risk asthma’ identified according to a validated algorithm. Staff in practices randomised to the intervention will complete two 45-min eLearning modules (an individually completed module giving background to ARRISA and a group-completed module to develop practice-wide pathways of care) plus a 30-min webinar with other practices. On completion of training at-risk patients’ records will be coded so that a flag appears whenever their record is accessed. Practices will receive a phone call at 4 weeks and a reminder video at 6 weeks and 6 months. Control practices will continue to provide usual care. We will extract anonymised routine patient data from primary care records (with linkage to secondary care data) to determine the percentage of at-risk patients with an asthma-related crisis event (accident and emergency attendances, hospitalisations and deaths) after 12 months (primary outcome). We will also capture the time to crisis event, all-cause hospitalisations, asthma control and any changes in practice asthma management for at-risk and all patients with asthma. Cost-effectiveness analysis and mixed-methods process evaluations will also be conducted.

**Discussion:**

This study is novel in terms of using a practice-wide intervention to target and engage with patients at risk from their asthma and is innovative in the use of routinely captured data with record linkage to obtain trial outcomes.

**Trial registration:**

ISRCTN95472706. Registered on 5 December 2014.

**Electronic supplementary material:**

The online version of this article (10.1186/s13063-018-2816-z) contains supplementary material, which is available to authorized users.

## Background

Worldwide, asthma affects 334 million people [1]. In the UK, which has one of the highest prevalence rates in Europe, there are 5.4 million patients with asthma, and asthma affects one in five households [2]. A quarter of all asthma patients have poor symptom control [3] and 185 people are admitted to hospital because of asthma attacks every day in the UK [2]. Asthma attacks result in major social, psychological and healthcare costs. For example, attacks are associated with a doubling of the healthcare costs for managing severe asthma in both children and adults [4, 5]. Asthma is, therefore, a common condition which results in a large, unnecessary personal, healthcare and economic burden [6]. Worryingly, given that there are an increasing number of effective treatments and long-standing, evidence-based management guidelines for asthma [7], the death rate for asthma has not reduced over recent years [8].

It is widely accepted that the majority of deaths and hospital admissions from asthma are associated with preventable factors [9]. In 2012, this prompted a National Review of Asthma Deaths (NRAD) [10], which showed that poor access to care and poor adherence with preventive medication(s) were key contributors in the deaths of a large proportion of patients. Patient-assessed poor access to primary care is also associated with emergency hospital admissions for asthma [11]. Unfortunately, due to complicating clinical and psychosocial characteristics, patients with asthma who are at particular risk of attacks (at-risk asthma) often fail to attend routine appointments or engage in initiatives [12], such as self-management education, that improve outcomes in general asthma populations [13]. In addition, these patients characteristically under-use primary care services, often fail to attend scheduled appointments [14, 15] and have difficulty adhering to treatments [16, 17]. It is these same patient groups that are also most often excluded from clinical trials. Existing evidence is thus unlikely to be generalisable [18] and research is urgently needed to identify better management strategies for this group. Systematic nationwide training and implementation programmes, mostly based in primary care, are able to reduce the morbidity and impact of asthma with reduced costs [19]. Also, a clinical care pathway for children with asthma has been shown to modify practice behaviour and reduce emergency department attendance and hospitalisations, but not significantly so, compared to usual care [20]. Indeed, the current British Thoracic Society/Scottish Intercollegiate Guideline Network (BTS/SIGN) British guideline on the management of asthma notes that ‘*well-conducted studies are needed to define the benefits of care pathways for asthma. These should include large, suitably powered studies to clarify the impact of pathways promoting systematic management of people with high-risk asthma in UK primary care’.* [7]

In an observational pilot study at a single primary care practice [21], electronic flagging of the electronic health records (EHRs) of 26 patients, demonstrating characteristics previously associated with adverse asthma outcomes, and training all practice staff in appropriate actions to take on seeing the flag was associated with a reduction in the number of emergency events for asthma in these individuals over 1 year, compared to 26 age-, sex- and treatment-matched controls. Our subsequent regional cluster randomised trial involving 29 primary care practices in Norfolk [22] covering 911 people at a high-risk of experiencing asthma crisis events (at-risk asthma, defined in line with contemporaneous guidelines as severe asthma plus adverse psychosocial factors) similarly flagged patients’ EHRs and delivered a 1-h practice-based training session for practice staff. The intervention did not reduce the primary composite endpoint of asthma attacks [23, 24] over 1 year. However, increases in the number of prescriptions of prednisolone (31% (95% confidence interval (CI) − 8 to 85)) masked reductions in the percentage of hospitalisations (50% (95% CI 6 to 74)) and accident and emergency (A&E) attendances (26% (95% CI − 31 to 58) amongst patients in intervention versus control practices. The intervention was well received by practice staff (according to an exit questionnaire) and resulted in an estimated cost-saving of £138.21 (95% CI £1248 to − £910) per patient in the intervention arm.

This regional study suggested that our primary care intervention for at-risk asthma patients (ARRISA) may have potential as a strategy for improving practice-level management and reducing emergency admissions, near-fatal asthma and asthma deaths, in this group. However, it remains to be seen whether these findings would translate into a meaningful clinical benefit at a larger scale. Furthermore, the identification of at-risk individuals in the previous studies was achieved by manually searching records to provide evidence of adverse psychosocial factors and the training was undertaken via a face-to-face, practice-based group discussion led by an experienced facilitator. These features of the intervention would be difficult to roll out nationally so we modified these processes in our current study: individuals with at-risk asthma will be automatically identified using a computer code containing details of an algorithm based on data routinely available in primary care EHRs and the training will be delivered via an online eLearning platform and webinars. Furthermore, in contrast to the original study where outcome data were obtained by semi-manually searching the primary care EHRs, in the current study we will utilise anonymous data extraction and record linkage of primary and secondary care records.

The study aims to test the hypothesis that systematically identifying patients at risk of severe exacerbations of asthma, flagging their primary care EHR to provide enduring prompts at the time of contacts with the practice, training all practice staff about the systematic management of these patients and providing on-going practice support (hereafter referred to as creation and integration of a primary care at-risk asthma register) will reduce crisis asthma events (asthma-related deaths, hospitalisations and A&E attendances) for at-risk patients and be clinically acceptable and cost-effective without detriment to the care of non-at-risk asthma patients.

Our intervention operates at the practice level. A cluster randomised trial design is, therefore, appropriate with the general practice as the unit of randomisation. As such, although the majority of objectives (including primary endpoint) and the health economic analysis pertain to the participant level, some objectives pertain to the cluster level (including aspects of the process evaluation). The study will be conducted in a controlled open study with blind assessment. The comparator will be usual care as defined by the current BTS/SIGN British Asthma Guideline [7] and National Institute for Health and Care Excellence (NICE) guidelines [25].

## Methods

### Aims

The primary aim of this study is to determine whether the creation and integration of at-risk asthma registers into primary care, as conceptualised in our ’At-Risk Registers Integrated into primary care to Stop Asthma crises’ (ARRISA) intervention, reduces asthma-related crisis events (A&E attendances, hospitalisations and deaths) for *at-risk patients* over a 12-month period compared to control practices. Our secondary aims are to assess outcomes (time to first crisis event, asthma control, all-cause admission or death) for *all* patients with asthma; and also to examine changes in the processes of asthma care (treatments, vaccinations, use of action plans or smoking cessation services, adherence to treatment) within practices. We also aim to assess whether this intervention is acceptable to healthcare professionals and patients and cost-effective from the perspective of the National Health Service (NHS). We will identify characteristics that influence uptake, integration and effectiveness of the intervention and explore causal mechanisms leading to changes in outcomes, including any unanticipated effects.

### Trial design

**Fig. 1:**
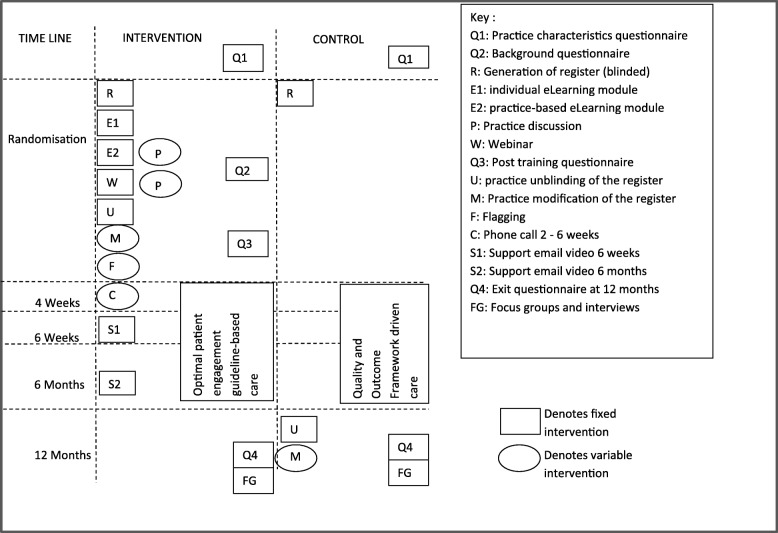
ARRISA-UK trial outline

**Fig. 2:**
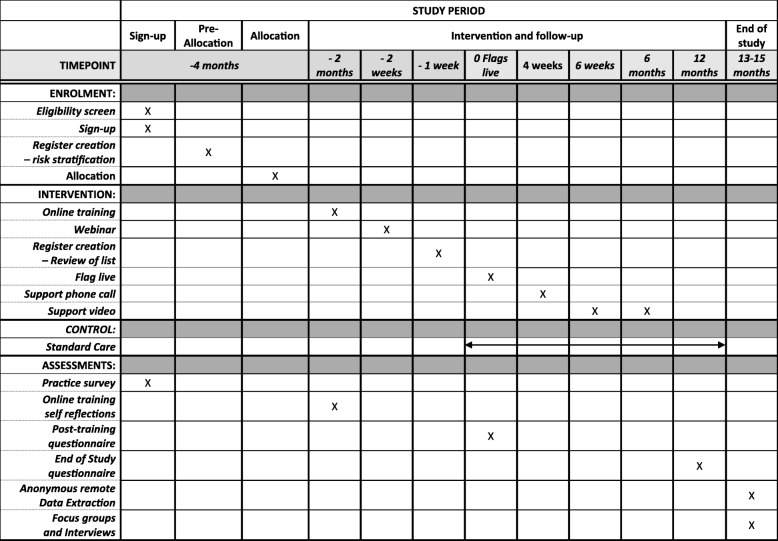
ARRISA-UK schedule of enrolment, interventions and assessments

This is a two-arm, pragmatic, multicentre, cluster randomised controlled trial plus health economic and process evaluation comparing usual care (control) to a complex, primary care practice-level intervention comprising: (1) creation of an asthma at-risk register, (2) online eLearning training for practice staff, (3) Computerised Decision Support System (CDSS) involving flagging of at-risk patients’ records to prompt agreed actions and (4) ongoing practice support. The intervention targets all healthcare professionals and reception staff within practices. The trial outline is shown in Fig. 1 and the schedule of enrolment, interventions and assessments is shown in Fig. 2. The trial protocol (v1.3, dated 23 March 2018) is based on the Standard Protocol Items: Recommendations for Interventional Trials (SPIRIT) 2013 Statement for protocols of clinical trials (see Additional file 1).

Routinely available data from patient records on outcomes, processes of care and healthcare use will be captured anonymously, without the involvement of the practice, at the end of the 12-month study period. Additional resource use data, for the purposes of economic evaluation, will be captured during the online training, post training and 12-month questionnaires. Data from a baseline practice demographic survey, the training software, questionnaires completed post training and at 12 months, plus focus groups and individual interviews undertaken with a sub-sample of practice staff and patients towards the end of the study, including those from the whole asthma population and those with at-risk asthma in the intervention and control groups (see below), will contribute to the process evaluation. This will take a mixed-methods sequential explanatory approach to exploring implementation, mechanisms and contextual influences on the intervention.

### Setting

The setting is primary care practices within the UK, with health economic analysis from the perspective of the NHS.

## Participants

### Clusters – Primary care practices

#### Recruitment

Primary care practices are being identified via the National Institute for Health Research (NIHR) Clinical Research Networks (CRN), Health and Care Research Wales, the Scottish Primary Care Research Network (SPCRN) and respiratory charities, societies and networks. The regions involved are: East Midlands, Eastern, Greater Manchester, Kent Surrey and Sussex, North East and North Cumbria, North Thames, North West Coast, North West London, South West Peninsula, Thames Valley and South Midlands, Wessex, West Midlands, West of England, Yorkshire and Humber in England, plus Scotland and Wales. Practices are selected by purposive sampling to ensure adequate representation from the devolved nations, from urban versus rural areas, and using different general practitioner (GP) software systems. All practices complete an online practice questionnaire as part of the recruitment process to check eligibility before randomisation and to provide stratification information and demographic data.

#### Inclusion criteria

Primary care practices in the UK.

#### Exclusion criteria

1. Practices already implementing a formal, prospective process for identification of patients with at-risk asthma and practice-wide targeting of asthma care on every contact with a patient or their record

2. Practices hosting or affected by research, or other aspects of care, which might significantly influence the practice wide process of care for patients with ‘at-risk’ asthma, or the ability to complete the study as planned; including incompatible practice software

Where decisions based on these criteria are not clear-cut, information about the practice circumstances is collected by the research team and a decision on eligibility taken by a panel of investigators (AW, HP, MN).

### Individual patients

Patients at recruited practices are included in the study if they have at-risk asthma as determined by our algorithm applied to routinely available primary care data forming the ‘original register’. Practice staff may add or remove patients from the register (see below), but the primary analysis will be conducted using data from all patients on the original register without additions or withdrawals. In addition, data from all patients with asthma defined on the basis of being on the practice asthma register will be included for the purpose of examining secondary outcomes.

Patients will be excluded if they have refused for their anonymous data to be used in research or are terminally ill and receiving palliative care. Patients have the right to refuse access to their data at any point until the conclusion of the data extraction.

### Process evaluation

#### Inclusion

As well as cluster- and patient-level data as above, for the purposes of the process evaluation selected practice staff and patients at participating practices will be approached to be involved in focus groups and/or qualitative interviews.

#### Exclusion

Individuals of under 16 years of age, unable to communicate in English or give informed consent will be excluded from focus groups and interviews.

### Intervention

This is a practice-wide (cluster-level) complex intervention comprising four main components:

1. Establishment of a practice at-risk asthma register:

We have developed and validated an algorithm, using routinely collected primary care data, for identifying those patients most at risk of attending A&E, being admitted to hospital or dying from an asthma attack. Factors in the algorithm include previous exacerbation history, coding for anxiety or depression, smoking history and prescribing data and laboratory results. Automated electronic searches identify patients with at-risk asthma from practice-based data using this algorithm. This is a two-stage process with the initial generation of an anonymised list and then coding to provide the names and details of the patients. Programmes and instructions for searches, with frequently asked questions, for the specific computer software programme of each practice will be sent to the information technology (IT) lead at the practice by email or compact disc (CD).

A representative (usually asthma GP lead or asthma nurse) at each intervention practice receives a document by email which explains ‘at-risk asthma’ and gives instructions on how to modify the list of patients with their records flagged (see CDSS below). GPs and asthma nurses are able to review the computer-generated list of at-risk patients, and exceptionally, with documented justification of the change, delete any who should not be on the list (e.g. receiving palliative care) and identify any other ‘at-risk’ patients known to them. They may modify the list, as above, for the duration of the study although the primary analysis will be based on data from patients included in the original register prior to modifications

2.  Practice-wide, online training (eLearning)

We have developed an innovative multi-component practice-wide intervention based on the material delivered in the face-to-face training developed for the prior regional study [22], but incorporating eLearning modules similar to those that have been previously shown to be successful in changing clinician behaviours [26]. Each practice is asked to nominate a representative from each staff discipline, e.g. nurse, GP, pharmacist (in dispensing practices) and receptionist (referred to collectively hereafter as ‘practice representatives’). A dedicated member of the practice staff (generally the practice asthma nurse) is then charged with championing the intervention at the practice (referred to hereafter as the practice champion) and ensuring dissemination via the practice representatives to maximise uptake. Practice representatives receive a document describing the purpose of the training and providing log-in details to the eLearning training resource. The eLearning modules have been developed in conjunction with Healthcare-Learning (www.healthcare-learning.com). They are hosted on the HealthCare-Learning secure website with links from the Asthma UK Centre for Applied Research website (www.aukcar.ac.uk). The main purpose of the education is to advise all practice staff on actions to take when presented with the electronic flag (see CDSS below) on the EHRs of an at-risk patient. It comprises two eLearning modules and a webinar:

(a) An individually completed module

This provides information on the national and patient perspectives of managing at-risk asthma, NRAD results [10], findings from the studies that were precursors to the ARRISA-UK study [21, 22], the flags to be added to patient records and discipline-specific training as to suggested actions to be undertaken on seeing the flag. The training uses a case scenario to highlight the possible benefits of the intervention and self-assessments to permit active learning and self-reflection. This module is to be undertaken by the practice champion and representatives and takes up to 45 min to complete.

(b) A practice-representative team completed module

This module is organised and chaired by the practice champion and attended by all of the practice representatives. The purpose of this module is to permit representatives of the practice team to collectively reflect on the material contained in the first module and encourage practice staff to agree on and document how the intervention will be implemented in their practice via a practice ‘action plan’. This covers the wording of the flag, proposed actions of each staff discipline in response to the flag, plans for including information in staff induction and disseminating to all staff, stance on informing patients about their at-risk status and a method for communicating with out-of-hours services. This module takes up to 45 min to complete

(c) A webinar

Ideally, within 8 weeks of completion of the second module, the practice representatives are asked to dial into a 30-min ‘webinar’ (online discussion) which reviews the learning objectives and outcome from the modules. It permits discussion within and between practices (in groups of between two and eight practices) participating in the study, allowing them to share experiences and ideas, and refine their practice implementation and dissemination plans accordingly. The webinars are being delivered by the GP lead who originally implemented an at-risk asthma register at his practice [21], developed and led the face-to-face training in the prior study [22], and was involved in developing the eLearning modules

3. CDSS

The CDSS consists of a flag which appears on the EHR whenever a patient on the at-risk asthma register makes contact with any member of the practice team and the clinical record is opened, and is designed to prompt actions as per the practice’s ‘action plan’ (see above). The nature of the flag is dependent on the software used to manage the practice’s EHRs and varies between pop-ups that actively need clearing from screen to yellow Post-it style notes remaining on screen. ARRISA is compatible with the Vision (In Practice Systems Ltd., London, UK), EMIS (EMIS Health, Leeds, UK) and SystmOne (The Phoenix Partnership (TPP), Leeds, UK) EHRs which account for more than 90% of primary care practices in the UK. The wording of the flag is chosen by the practice during the second training module but suggested wording of ‘At-Risk Asthma Prioritise Care’ is given. Instructions for generating the flag are provided to the practice manager/IT lead. The flagging goes live after the completion of the minimum training as defined below

4. Practice support

The practice champion receives a phone call approximately 4 weeks (between 2 and 6 weeks) after the activation of the flags to ensure that there are no technical or other fundamental implementation issues. Practice representatives receive emails containing links to brief videos at 6 weeks and 6 months that remind practices of the purpose of the study and the importance of targeting, prioritising access for, engaging with, and effectively managing, patients with at-risk asthma at every opportunity.

Study newsletters will be circulated to practice representatives for the duration of the study. These provide updates on study progress including number of recruited sites, timelines, etc. In addition, practice staff have access to a helpline manned by an un-blinded IT technician for help with technical issues regarding the software for the duration of the study. The eLearning website contains links to national guidelines and other resources.

Full practice engagement with the training is encouraged as the flag is only activated after the completion of minimum training, which we define as at least one practice member completing both eLearning training modules. If required, we will send reminders to all practice representatives by email on two occasions then contact them by phone to encourage completion of the training. Assessment of engagement with the intervention will form part of the process evaluation and include examination of modifications to the register, uptake and completion of the training and engagement with follow-up phone calls.

### Control

Control practices produce an anonymised list of patients who meet the definition of at-risk asthma from the algorithm. They are given instructions and asked to enter a research-relevant Read code onto patients’ EHRs so that they can be identified for the purpose of data collection. However, practice staff are blind to the names and details of the patients on the list until the end of the study. The practice representatives from the control practices will also receive study newsletters for the duration of the study.

The control practices continue to provide their usual care. This may vary between practices, but reflects the recommendations of the BTS/SIGN British Asthma Guideline [7] and NICE guidelines [25]. Primary care practices in England and Wales are incentivised to provide care to BTS/SIGN standards as defined by the Quality and Outcome Framework (QOF) [27]. This recommends creating a register of all patients with asthma and offering at least annual practice-based asthma reviews (typically in nurse-led clinics). Recognised management includes checking control of asthma symptoms with the Royal College of Physicians’ three questions (RCP3Qs; i.e. difficulty sleeping, daytime symptoms, interference with usual activities), checking smoking status and offering cessation advice, assessing/teaching inhaler technique, assessing and offering advice on adherence, and delivering patient self-management education which may include provision of action plans and self-monitoring tools [28]. Follow-up in secondary care outpatient clinics and use of emergency primary and care services is available as usual for patients, if required.

After completion of the trial, control practice staff will be permitted to undertake the eLearning training and un-blind and modify the register in the same way as intervention practices could at the beginning of the study.

### Outcomes

#### Primary outcome

The percentage of at-risk patients with an asthma-related (defined as asthma, mixed asthma/chronic obstructive pulmonary disease (COPD) and/or mixed asthma/respiratory infections) crisis event – i.e. an A&E attendance, hospitalisation or death – over 12 months.

#### Secondary outcomes

1. The time to first, and rate of, asthma-related crisis event(s) (defined as above) for at-risk patients
2. The percentage of all registered asthma patients with an asthma-related crisis event, their rate of, and time to, first asthma-related crisis event(s) (as above)
3. The percentage of at-risk and all registered asthma patients with good control defined as answering ‘no’ to all of the RCP3Qs [29]
4. The percentage of at-risk and all registered asthma patients requiring a hospital admission or dying for any reason
5. The number of the following (per patient per year) for both at-risk and all registered asthma patients:

(a) short-acting bronchodilator prescriptions issued

(b) prescriptions of systemic corticosteroids for asthma attacks and antibiotic-treated lower respiratory tract infections

(c) modifications of the prescription of asthma-related medications to align more closely with current guidelines (e.g. increased use of inhaled corticosteroids)

(d) records of written personalised asthma action plans and patient self-monitoring with peak flow diaries

(e) inhaler technique assessments recorded

(f) smoking cessation advice or smoking cessation medications given

(g) flu vaccinations

(h) ‘did not attends’ at primary and secondary care routine appointments

(i) adherence to medication determined from validated computer-based calculations using prescription data

Health economic outcomes

6. The estimated (per patient) mean incremental cost of the at-risk register compared with usual care
7. The incremental effect of the at-risk register, in terms of the estimated (per patient) mean difference (between arms) in the number of asthma crisis events

#### Process evaluation outcomes

The outcomes include quantitative and qualitative assessments of the views of healthcare professionals and patients, practice procedures and processes of care, staff awareness and behaviours, and indicators of uptake, implementation and engagement with the intervention. The process evaluation will also explore how key intermediary and primary and secondary outcomes (e.g. asthma control) are affected by contextual characteristics (e.g. practice characteristics), including identification of factors that will improve effectiveness and sustainability in practice.

#### Safety outcomes

There will be no documentation or reporting of non-serious adverse events. Deaths and hospitalisation are the primary effectiveness outcome and will not be reported as safety measures. Practices will be asked to capture complaints or important study-related adverse events by completing a complaints/adverse events form and forward it to the Norwich Clinical Trials Unit (NCTU).

### Data collection

#### Baseline characteristics and outcome data

All practices expressing interest in the study are asked to complete an initial questionnaire to provide basic data on practice characteristics.

All participating practices must agree to share their practice EHRs with Optimum Patient Care (Cambridge, UK). Obtaining data in this way not only permits linkage to different databases (practice EHRs, Office for National Statistics (ONS), Hospital Episode Statistics (HES)), but also permits data capture without burdening the practice or patients. All practices (control and intervention) are asked to insert a specific Read code that cannot be readily removed by practice staff into the EHRs of at-risk asthma patients to denote inclusion in this study. This will be used by data managers and statisticians to identify these patients and distinguish their data from other patients with active asthma in the practice. For the purposes of data collection, active asthma is defined using the QOF criterion [27]: Read code for asthma diagnosis plus asthma therapy in the past year, excluding those coded as asthma resolved but not excluding QOF exception codes. At the end of the study, data extractions will be obtained for all patients with asthma in the practice containing data for the 12 months before randomisation and the 12 months following the activation of the flag in the intervention practices. The mean time from randomisation to activation of the flag will be calculated for all intervention practices, and data collection will be captured for 12 months from this time after randomisation in the control practices. For clinical event data and for patients’ demographic and other characteristics (weight, height, smoking status, etc.) the last recorded value will be obtained.

#### Health economic data

Resources associated with establishing the at-risk register, training, the CDSS and practice support will be estimated from questionnaires and data obtained from the training website. Additionally, details of patients’ NHS resource use will be extracted from routinely recorded data, where this will include all contacts with health professionals and hospital admissions and asthma-related medications.

#### Process evaluation data

Data captured from the training website for the purpose of process evaluation include module completion rates, time taken to undertake the training, time that the training took place, the number of ‘hits’ on the website resource pages, and changes in knowledge, attitudes and motivation during the training as indicated by self-assessment answers. Likewise, data collected from the helplines and phone calls to practices conducted between 2 and 6 weeks capture details of problems with the intervention and practice engagement. During the second training module, practice teams will be asked to complete online action plans, and these will be captured along with notes from the webinar facilitator, and recordings of webinars to assess details of, and variation in, practice-level dissemination and implementation of the intervention.

After the training, all practice representatives from intervention practices are asked to complete an online questionnaire to provide feedback regarding their experiences and the perceived impact of the training and flags. This will supplement the data captured by the training software and assess the acceptability of the intervention, further changes in knowledge, attitudes and motivation, etc. over time and potential mechanisms by which the training and flags may impact on processes of care. At the end of the study all practice representatives from the intervention practices will be asked to complete a further online questionnaire to provide information regarding their experience of the intervention and its integration into routine practice. ARRISA-UK practice champions from intervention practices and practice contacts from control practices will also be asked questions about major changes in practice asthma management policies over the preceding 12 months.

Taking a sequential, explanatory approach, findings from analyses of preliminary quantitative data will be used to inform sampling and topic guides for focus groups and interviews with relevant stakeholders towards the end of the study. These will explore, in more depth, causal mechanisms, impacts and any unintended consequences of the intervention, the acceptability of the intervention and contextual factors affecting implementation. It is anticipated that dual moderator focus groups [30] or interviews, will be undertaken with representatives from at least six practice/staff group teams (approximately eight participants per focus group) across several study regions. Practice staff and patients at participating practices will be identified and approached via the practice champion. Both patients and practice staff will be consented by process evaluation researchers prior to being involved in focus groups and/or qualitative interviews. A maximum diversity sample of participants will be recruited to reflect a range of engagement with the intervention/training and practice and staff characteristics, guided by quantitative data if this identifies issues for particular staff groups or types of practice. Each focus group will last approximately 1 h and will be digitally audio-recorded, then transcribed verbatim. A further three focus groups, using the same methodology, will be undertaken with patients from three of the practices represented in staff focus groups. The purpose of these focus groups will be to explore patients’ experiences and perspectives on the strengths and limitations of ‘at-risk’ registers and their potential for impact on patients’ asthma management. Fewer focus groups and interviews will be undertaken if data saturation becomes apparent. Information from early focus groups or interviews may also inform refinements to the end-of-study questionnaires used to collect quantitative data on the consequences and integration of the intervention into routine practice from across a broad range of practices.

### Data management

Anonymised outcome and healthcare resource use data will be received from Optimum Patient Care (routine primary care data), the Healthcare-Learning eLearning module database (training data), questionnaires and transcripts of focus groups and interviews. These will be uploaded onto a central study database stored on the servers based at University of East Anglia (UEA). The database will be password protected and only accessible to members of the ARRISA-UK trial team at NCTU, external regulators and process evaluation teams. The server is in a secure room, which is protected by closed-circuit television, where access is restricted to members of the UEA Information Systems team by security door access. The study database has been built using Microsoft Structured Query Language (SQL) Server tools and all Internet traffic is encrypted via an Hypertext Transfer Protocol Secure (HTTPS) connection, using Transport Layer Security (TLS) v1.2 and RSA 4096.u. periodically and at database lock the data will be checked for errors and inconsistencies. The database is designed to comply with the principles of the International Conference on Harmonisation (ICH) Good Clinical Practice (GCP), within the Standard Operating Procedures (SOP) for Data Management in NCTU and also where appropriate with UEA IT procedures. The database and coding values have been developed by the Head of Data Management in conjunction with the study statistician and other NCTU members.

### Sample size

A total sample size of 8204 patients from 235 practices will provide 90% power to detect a difference in the primary outcome (asthma crisis events) from 7% to 5% (effect size of 0.3) assuming a cluster size of 35 and intraclass correlation coefficient (ICC) of 0.01. These estimates are from the event rate and prevalence of predicted hospitalisations and A&E attendance, as a whole and per practice, obtained from a database of 48,000 patients obtained from Optimum Patient Care, and the cluster size and ICC from our regional trial [22]. The average cluster size was estimated from CRNs. Patients will, as noted above, only be removed from the register in exceptional circumstances and we estimate that less than 10% of patients will leave their practice [31]. Therefore, we planned to evaluate a total of approximately 9170 patients with at-risk asthma from 262 practices. However, the initial average cluster size (39 patients per cluster) was greater than estimated and we propose to increase the sample size to 270 practices with 10,530 patients. If more than 10% of practices do not undergo minimum training and flagging, additional practices will be recruited. A decision to stop the study will be considered if the power of the study is reduced to less than 80% or when more than 30% of practices fail to undergo training to a minimal degree and/or do not activate the flag.

### Randomisation

Practices will be randomised to the intervention or control, in a 1:1 ratio, based on a computer-generated randomisation code, prepared by the study statistician, with stratification for practice software (Vision/EMIS/SystmOne) and presence of an asthma-diploma-trained nurse at the practice (yes/no) at randomisation. Emails are sent to practice managers and practice champions informing them of their allocation with appropriate log-in details for the study website and, for the intervention practice, the eLearning modules.

### Blinding

This pragmatic cluster randomised controlled trial is being conducted with blind assessment and analysis of outcomes. The practices (clusters) cannot be blind to the intervention. The patients with asthma may or not be blind to the intervention depending on whether individual practices decide to inform patients they are on an ‘at-risk’ register. As far as possible, those researchers not interacting with the practice sites will remain blind throughout the study and analysis. The statistical analysis team will be blind to the intervention throughout. The health economists will be blind during their initial determination of costs and until that point in the analysis plan where they must include costs associated with the allocation. The researchers implementing the intervention, including the primary care physician leading the webinar training programme, the qualitative interview and focus group researchers and the research trial staff providing assistance on practice sign up, follow-up phone calls and providing support for the software, are, of necessity, aware of allocation.

### Analysis

#### Effectiveness analyses

The primary comparison of treatment arms at 12 months will be based on the intention-to-treat (ITT) principle including all patients initially identified with ‘at-risk’ asthma using the risk algorithm. The primary outcome will be analysed on an individual-level using a logistic regression model with random effects to allow for the clustering of patients by practice and fixed effect for the factors included in the stratification, i.e. practice software and practice diploma-trained nurse. A similar model will be used for the number of events, based on a Poisson regression model with random and fixed effects, and the time until the first event. Secondary outcomes will be analysed in a similar fashion, which is to say that the binary outcomes will be analysed using logistic regression models and all count outcomes using Poisson regression. All model assumptions will be assessed using appropriate techniques and for Poisson regression models over dispersion will also be checked.

#### Additional subgroup analyses

As well as the above ITT analyses (of data from patients on the register at the time of randomisation) some further pre-planned analyses will be conducted. In particular: (1) restriction of the analyses to only those patients who remain on the register over the course of the year (unless removed due to death); (2) restriction of the analysis to those intervention practices where all staff representatives completed over 80% of the training; and (3) inclusion of those individuals subsequently added to the ‘at-risk’ register by the practice.

#### Health economic analyses

In line with the primary analysis, the health economic base-case analysis will be conducted on an ITT basis. Appropriate unit costs [32] will be assigned to each item of resource use for a standard price year. The incremental cost of the at-risk register compared with standard NHS care without an at-risk register, over the 12-month trial period, will then be estimated by comparing the mean overall cost (to the NHS) in each arm of the study (with adjustment for other factors, e.g. clustering). Similarly, the incremental effect will be estimated by comparing the mean number of asthma crisis events between arms. Additionally, based on data from other sources, we will attempt to assign an estimated loss in quality of life to each asthma-related crisis event, in order to enable the incremental effect to also be estimated in terms of Quality-adjusted Life Years (QALYs).

The incremental cost and incremental effect will subsequently be used to estimate the cost-effectiveness of the at-risk register. If dominance occurs, where the at-risk register is both associated with a lower (higher) cost and is more (less) effective, then the at-risk register would be estimated to be cost-effective (or not). Alternatively, the incremental cost-effectiveness ratio (ICER) [33] will be estimated by dividing the incremental cost by the incremental effect, where this will be compared to any relevant thresholds, e.g. [34] in order to assess value for money.

Non-parametric bootstrapping will be used to explore the level of uncertainty surrounding the cost-effectiveness results, and to construct cost-effectiveness acceptability curves [35]. Sensitivity analysis [33] will also be undertaken to assess the robustness of conclusions to changes in key assumptions, including the aforementioned additional analyses.

#### Process evaluation analyses

**Fig. 3:**
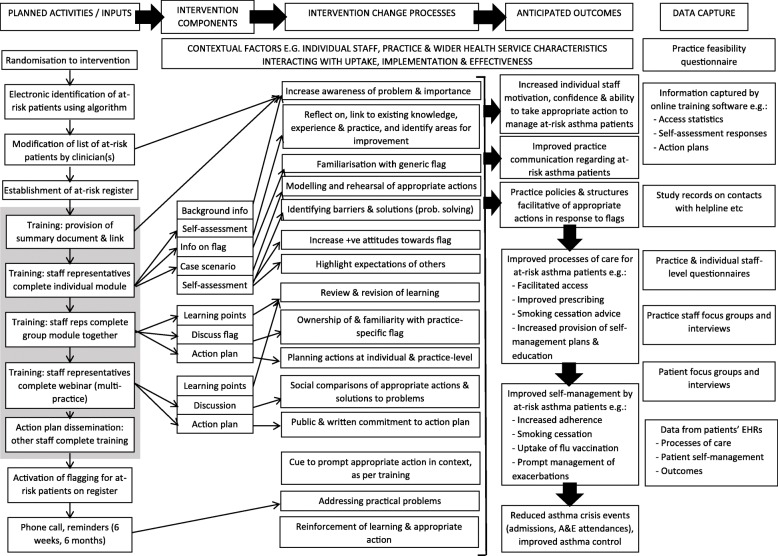
ARRISA-UK process evaluation logic model

A comprehensive, mixed-methods process evaluation in line with the Medical Research Council (MRC) guidance [36], a published framework on the conduct of process evaluation alongside cluster trials [37] and initial logic model of the intervention (Fig. 3) will be undertaken. Using the mixed-methods sequential explanatory approach [38], descriptive summaries of initial quantitative data will inform the focus of later qualitative research, and any novel findings from the qualitative research will generate further hypotheses for exploration with analyses of quantitative data where possible. The influence of selected moderating contextual factors (e.g. practice characteristics) on intermediary (e.g. process of care) and clinical outcomes (e.g. crisis events, asthma control) will be explored using regression models. Regression models may also be used in mediation analyses to assess potential mechanisms of the intervention.

Analyses of qualitative data from focus groups and interviews will be undertaken using Framework Analysis [39] and supported by use of NVivo 9.0 software. At least two project team members will analyse each transcript to enhance rigour. Efforts will be made to integrate qualitative and quantitative process evaluation and outcome data where possible to provide triangulation and aid interpretation of findings, e.g. using matrix approaches.

### Ethical issues and oversight

The study is being conducted in accordance with CODEX rules and guidelines for research and the Helsinki Declaration as well as the International Conference on Harmonisation (ICH) Guideline for Good Clinical Practice (GCP). The study protocol was approved by North Wales Research Ethics Committee (reference 14/WA/1211) prior to the start of the study. The study is registered on the International Standard Randomised Controlled Trials Number (ISRCTN) registry (reference ISRCTN95472706). Approval was granted by the Health Research Authority (HRA) and Confirmation of Capacity and Capability to conduct the study has been provided by primary care R&D offices (including local CRN) and/or individual practices as relevant.

Practices managers are asked to sign a contract with UEA on behalf of all practice staff. Patients will not be required to provide consent to use their data as their extracted data are anonymised. Asthma is a common condition and, therefore, patients are not potentially identifiable. All practices are asked to ensure that their patients are aware (e.g. by displaying a poster, statements on websites) that their data may be used anonymously for research as this is standard procedure in research-ready practices. The Read code identifying those patients that are at-risk remains on the practice systems and only non-identifiable data will be extracted. Quotes from patients at focus groups or interviews that are published will be anonymised. The focus group and interviews will not be exploring highly sensitive issues and, therefore, the study has no material ethical issues; however, individuals participating in the focus groups or interviews will provide written informed consent before participating.

UEA is the trial sponsor and has delegated responsibility for the overall management of the trial to the chief investigator and NCTU including the trial design, coordination, monitoring and analysis and reporting of results. The standard procedures and policies at NCTU, a UK Clinical Research Collaboration (UKCRC)-registered trial unit and the study’s Quality Management Monitoring Plan are followed. A Trial Management Group (TMG) has been set up to assist with the design, coordination and strategic management of the trial. An independent Trial Steering Committee (TSC) and independent Data Monitoring Committee (DMC) have also been set up to provide oversight on all aspects of the trial and to safeguard the interests of primary care practices and patients, including review of protocol amendments. Both the TMG and TSC have lay membership.

#### Dissemination

We will publish the results of the study in peer-reviewed journals and present the data at national and international conferences. Asthma UK, the Asthma UK Centre for Applied Research (AUKCAR) and our hospital and university media relations departments pledge to support dissemination of the study outcomes with coordinated press release and media coverage. We will use the innovative dissemination channels of the AUKCAR (websites, public lectures and blogs) and the co-applicants professional networks to ensure that findings are both disseminated and adopted into practice if the intervention is found to be effective. Asthma UK will support dissemination to patients through their network of volunteers and members, and to professionals via their Healthcare Professional Relationships Manager.

Results will be reported following the use cluster extension to the Consolidated Standards of Reporting Trials (CONSORT) and the REporting of studies Conducted using Observational Routinely collected health Data (RECORD) statements.

## Discussion

This protocol describes a study that will explore the impact of a primary care practice-wide intervention designed to improve processes of care for at-risk asthma. It is based on a previous regional study, with modifications (automatic at-risk patient identification, online training and remote anonymous data capture) which permit large-scale implementation within a UK-wide multicentre study across a large number of diverse practices.

Our intervention comprises automatic generation of a list of people with at-risk asthma using a validated algorithm based on data available from primary care EHRs that are used by more than 90% of primary care practices in the UK. This permits standardised participant identification throughout the country and also between intervention and control practices. However we permit modification of the list of at-risk patients for use by the practice as we anticipate that clinical knowledge of their patients will allow refinement of the list. In addition, patients’ at-risk status is likely to change over time. We have also automated the training aspect of the intervention so that it is delivered via an online eLearning package plus a webinar. This means that the intervention could be rolled out throughout the UK and adopted into clinical practice quickly if our intervention is deemed beneficial. In this respect, a primary-care-based intervention, which included education and asthma-nurse-led patient reviews with an emphasis on self-management plans, has previously been shown to increase in the time to first attendance with acute asthma from 126 to 194 days (HR 0.73 (0.54–1)) [40], as do interventions which utilise a practice champion [40–42]. Our intervention is encouraged by a practice champion and individuals are reminded of the training via videos sent at 6 weeks and 6 months and also every time the flag appears.

We have designed the ARRISA-UK CDSS to maximise its impact. The CDSS is a single flag which appears automatically whenever the patients’ EHRs are opened (and is, therefore, timely) and it cannot be readily switched off by practice staff (it is, therefore, inescapable). The precise nature of the flag varies somewhat between practice software (which is one of the reasons that the study is stratified for practice software) and varies from a pop-up box to one that has to be cleared before any further action can be taken. The wording of the flag is agreed by the practice representatives at the second eLearning module (the group-based session where practice-wide action plans are agreed) and can be changed during the study, if required. The flag is designed to prompt actions as agreed in the practice action plan disseminated to all staff, so allows tailoring to practices’ existing processes of care.

The data required for the primary and secondary care outcomes will be obtained from anonymous extraction of routine healthcare data, without obtaining individual informed consent as has been the case in similar studies [22, 26, 31, 43]. However, we ask all practices to inform patients that their data may be used anonymously for research and practice managers will be asked to sign a contract with UEA on behalf of all practice staff. Written informed consent will be obtained from patients to participate in focus groups or interviews. We believe that this meets ethical standards as the intervention is at practice level and it is the practice staff/infrastructure that are directly involved, not the patients. The Read code identifying patients remains on the practice systems and only non-identifiable data will be extracted. The data extraction methods meet all legal requirements (http://optimumpatientcare.org/about-us/) and using anonymous patient data in this way is a recognised strategy to improve patient care in line with the government agenda including the care.data initiative (http://www.england.nhs.uk/ourwork/tsd/care-data/).

### Trial status

The current version of the protocol is version 1.2, 4 February 2018. The trial began in August 2015 and we expect to complete recruitment in April 2018.

## Additional file

Additional file 1: Standard Protocol Items; Recommendations for Interventional Trials (SPIRIT) 2013 Checklist: recommended items to address in a clinical trial protocol and related documents. (DOC 121 kb)

## Abbreviations

A&E: Accident and emergency
AUKCAR: Asthma UK Centre for Applied Research
BTS: British Thoracic Society
CD: Compact disc
CDSS: Computerised Decision Support System
CI: Confidence interval
CONSORT: Consolidated Standard of Reporting trials
COPD: Chronic obstructive pulmonary disease
CRN: Clinical Research Network
DMC: Data Management Committee
EHR: Electronic health record
GCP: Good Clinical Practice
GP: General practitioner
HES: Hospital episode statistics
HRA: Health Research Authority
HTA: Health Technology Assessment
HTTPS: Hypertext Transfer Protocol Secure
ICC: Intraclass correlation coefficient
ICER: Incremental cost-effectiveness ratio
ICH: International Conference on Harmonisation
IT: Information technology
ITT: Intent-to-treat
MRC: Medical Research Council
NCTU: Norwich Clinical Trials Unit
NHS: National Health Service
NICE: National Institute for Health and Care Excellence
NIHR: National Institute for Health Research
NRAD: National Review of Asthma Deaths
ONS: Office for National Statistics
QALY: Quality-adjusted Life Years
QOF: Quality and Outcomes Framework
RCP3Q: Royal College of Physicians three Questions
REC: Research Ethics Committee
RECORD: REporting of studies Conducted using Observational Routinely collected health Data
SIGN: Scottish Intercollegiate Guideline Network
SOP: Standard operating procedures
SPCRN: Scottish Primary Care Research Network
SQL: Structured Query Language
TMG: Trial Management Group
TPP: The Phoenix Partnership
TSC: Trial Steering committee
UEA: University of East Anglia
UK: United Kingdom
UKCRC: UK Clinical Research Collaboration
